# *Ex Vivo* Test for Measuring Complement Attack on Endothelial Cells: From Research to Bedside

**DOI:** 10.3389/fimmu.2022.860689

**Published:** 2022-04-12

**Authors:** Marie-Sophie Meuleman, Anna Duval, Véronique Fremeaux-Bacchi, Lubka T. Roumenina, Sophie Chauvet

**Affiliations:** Centre de Recherche des Cordeliers, INSERM, Sorbonne Université, Université de Paris, Paris, France

**Keywords:** complement, endothelial cells, diagnostics, prognostics, therapeutics, kidney injury, nephrology, explorations

## Abstract

As part of the innate immune system, the complement system plays a key role in defense against pathogens and in host cell homeostasis. This enzymatic cascade is rapidly triggered in the presence of activating surfaces. Physiologically, it is tightly regulated on host cells to avoid uncontrolled activation and self-damage. In cases of abnormal complement dysregulation/overactivation, the endothelium is one of the primary targets. Complement has gained momentum as a research interest in the last decade because its dysregulation has been implicated in the pathophysiology of many human diseases. Thus, it appears to be a promising candidate for therapeutic intervention. However, detecting abnormal complement activation is challenging. In many pathological conditions, complement activation occurs locally in tissues. Standard routine exploration of the plasma concentration of the complement components shows values in the normal range. The available tests to demonstrate such dysregulation with diagnostic, prognostic, and therapeutic implications are limited. There is a real need to develop tools to demonstrate the implications of complement in diseases and to explore the complex interplay between complement activation and regulation on human cells. The analysis of complement deposits on cultured endothelial cells incubated with pathologic human serum holds promise as a reference assay. This *ex vivo* assay most closely resembles the physiological context. It has been used to explore complement activation from sera of patients with atypical hemolytic uremic syndrome, malignant hypertension, elevated liver enzymes low platelet syndrome, sickle cell disease, pre-eclampsia, and others. In some cases, it is used to adjust the therapeutic regimen with a complement-blocking drug. Nevertheless, an international standard is lacking, and the mechanism by which complement is activated in this assay is not fully understood. Moreover, primary cell culture remains difficult to perform, which probably explains why no standardized or commercialized assay has been proposed. Here, we review the diseases for which endothelial assays have been applied. We also compare this test with others currently available to explore complement overactivation. Finally, we discuss the unanswered questions and challenges to overcome for validating the assays as a tool in routine clinical practice.

## Introduction

As part of the complex innate immune surveillance system, the complement system plays a key role in defense against pathogens and in host homeostasis. This enzymatic cascade is rapidly triggered in the presence of activating surfaces, such as bacteria or apoptotic necrotic cells. However, the cascade is highly physiologically regulated on host cells to avoid self-aggression. The endothelium is one of the primary targets of complement dysregulation. There is increasing evidence of complement implications in the pathophysiology of many human diseases. Many complement-blocking therapeutics are under development, and some are already available in clinical practice. Nevertheless, detection of abnormal functioning complement is challenging, because in many pathological conditions C3 and C4 plasma levels, the two main biomarkers of complement activation, remain within normal ranges. The available tests to demonstrate such overactivation with diagnostic, prognostic, and therapeutic implications are limited. Methods are poorly standardized, and only a few have functional value. Therefore, there is a need to develop a robust and standardized tool for identifying infraclinical complement activation.

The final objective is to allow better pathophysiologically based therapeutic management of patients. The analysis of complement deposits on cultured endothelial cells (EC) incubated with patient serum holds promise as a reference assay. This approach has been used to explore complement activation in the sera of patients with atypical hemolytic uremic syndrome (aHUS), malignant hypertension, hemolysis, elevated liver enzymes, and low platelet (HELLP) syndrome, sickle cell disease (SCD), and pre-eclampsia. In some cases, adjusting the complement-blocking drugs has been considered. Nevertheless, the international standard for this test is lacking, and the mechanism by which complement is activated in this assay is not fully understood.

After a brief summary of the complement cascade, we present the mechanisms of complement activation and how they contribute to cell damage in several human diseases. We then provide an overview of the tests currently available to explore complement overactivation in routine practice. Finally, through a comparative analysis of the available endothelial assays for complement exploration, we discuss the unanswered questions and challenges to overcome to validate the study of complement deposition on cultured EC as a tool in routine clinical practice.

## The Complement System in Health and Disease

The complement system plays a key role in cell homeostasis, inflammation, and defense against pathogens. It is the first line of defense. The system comprises more than 30 soluble and membrane-bound proteins. Three different pathways lead to complement activation: the classical (CP), lectin (LP), and alternative (AP) pathways. When activated, these serine protease cascades converge to the formation of two enzymes, C3 convertase and C5 convertase, allowing the generation of the main effectors of this system: anaphylatoxins (C3a and C5a), opsonin (C3b/iC3b), and the membrane attack complex (MAC) (C5b-9). CP and LP are initiated by the recognition of pathogen-associated molecular patterns or damage-associated molecular patterns by pattern-recognition molecules (C1q and mannose-binding lectin). Conversely, AP is constantly activated at a low level in the fluid phase, generating a small quantity of C3b. In the presence of an activating surface (apopto-necrotic or bacterial), C3b covalently binds to the surface, and thus, initiates cell surface C3 convertase formation (C3bBb) and the AP amplification loop. To avoid self-aggression, AP is highly regulated in the fluid phase and on the host cell surface by soluble (factor H (FH), factor I (FI)) and membrane-bound regulators (membrane cofactor protein (MCP) or CD46, complement receptor 1 (CR1) or CD35, decay accelerating factor or CD55, and CD59). In humans, deficiencies in complement regulatory proteins are associated with rare diseases, such as aHUS, C3 glomerulopathy (C3G), and paroxysmal nocturnal hemoglobinuria (PNH). However, complement activation triggered by different pathophysiological processes that overwhelm the capacity of regulation has been increasingly described in a wide spectrum of diseases.

## Complement Implication in Diseases

**Figure 1:**
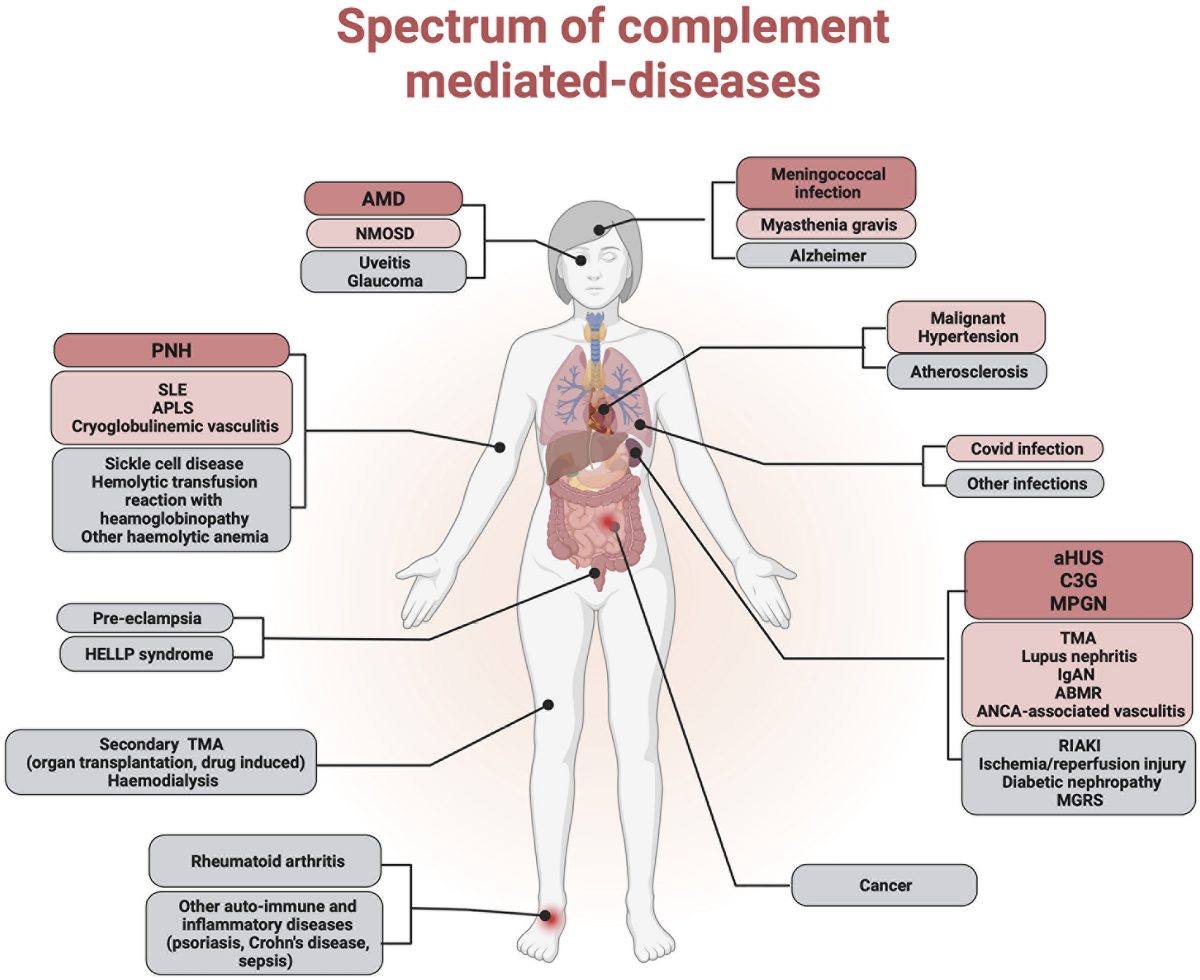
Complement implication in human diseases. Complement dysregulation has been implicated in the pathophysiology of several human diseases. Complementopathies in which alternative pathway dysregulation is the central mechanism of cell and tissue injury are represented in red. Conditions in which the complement system has been demonstrated to contribute significantly to tissue injury are represented in pink. Other diseases in which complement plays an accessory role are represented in gray.

While AP overactivation is the central mechanism of cell and tissue injury in complementopathies (aHUS, C3G, and PNH), complement is crucial to tissue injury in a wide variety of diseases. These include age-related macular degeneration (AMD), antibody-mediated rejection (ABMR), cryoglobulinemic vasculitis (CV), IgA nephropathy (IgAN), systemic lupus erythematosus (SLE), anti-phospholipid syndrome (APS), ANCA-associated vasculitis (AAV), rheumatoid arthritis (RA), HELLP syndrome, pre-eclampsia, myasthenia gravis (MG), neuromyelitis optica spectrum disorder (NMOSD), SCD, and rhabdomyolysis-induced acute kidney injury (RIAKI). To a lesser extent, complement seems to be involved in an increasing spectrum of human pathological conditions, such as inflammatory disorders, ischemia/reperfusion, cancer, degenerative disorders (e.g., Alzheimer’s disease, atherosclerosis), and more recently, viral infections that include COVID-19 (1) (**Figure 1**).

Complementopathies are characterized by a specific cell target of AP-mediated damage. In aHUS and PNH, AP dysregulation occurs on the cell membrane, EC surface or platelets (2) and erythrocyte surface (3). In C3G, overactivation of C3 and C5 convertases may occur in the fluid phase or locally within the glomeruli, where the targeted surface remains to be determined (suggestions include glomerular EC and mesangial cells). AP dysregulation is a central pathophysiological mechanism in these diseases. It can be related to innate or acquired abnormalities in complement components, mainly regulators (FH, FI, or MCP) or C3 convertase components (C3 or FB) (4–15).

In diseases with major complement contributions, complement activation can be triggered by one or another pathway. In CV (16) and ABMR (17), activation occurs through CP in the presence of immune complexes (IC). In cryoglobulinemia (type II), IC are composed of IgM with rheumatoid factor activity associated with polyclonal IgG. In ABMR, IC are composed of IgG and donor HLA molecules. Conversely, despite the disease being triggered by the presence of IC, AP appears to be essential for disease development in mouse models of RA (18, 19) and SLE (20–22). This activation can be enhanced by apoptotic and necrotic cells due to prior damage (23) or by proteins of the extracellular matrix (ECM) from damaged cartilage in RA (24). In IgAN, AP (25), and LP (26) activation is mediated by polymeric IgA. *In vitro*, a correlation was found between C3 cleavage products (iC3b, C3c, C3dg) and IgA-A-IgG IC levels, suggesting that IC-containing IgA may act as a surface for soluble AP activation (27). In AAV, AP may be activated by neutrophil extracellular traps, thus amplifying complement activation and damage of EC (28). Finally, a disease-specific soluble factor has been implicated in complement activation. Free heme renders EC more sensitive to complement activation in SCD (29), aHUS (30), and RIAKI (31). *In vitro*, thrombin induces C5 cleavage in C5a in APS (32).

Complement activation does not arise from a unique mechanism but can be triggered in several ways according to the disease pathophysiology. Identification of the precise mechanisms of complement activation will help determine different potential therapeutic targets within the cascade.

Complement activation contributes to cell and tissue injuries in different ways. First, it promotes inflammatory cell recruitment mainly in CV (33), ABMR (34), AMD (35), SLE (36)and RA. C5a and its receptor C5aR are involved in neutrophil recruitment (37–39) and endothelial activation (40) in AAV. Complement activation can promote specific disease processes. Thus, MAC can directly affect collagenase production by synovial fibroblasts in RA (41). In IgAN, mesangial cells exposed to complement activation and C3 deposition promote phenotypic conversion to a more synthetic and proliferative state (42). In AMD, C3a and C5a promote choroidal and C5a induces vascular endothelial growth factor secretion by retinal pigment epithelium (35). In pre-eclampsia, it has been suggested that the binding of C5a to C5aR expressed on trophoblasts contributes to the acquisition of their anti-angiogenic phenotype (43).

The complement system can also act as an amplifier for other molecules involved in injury. The C5a/C5aR axis participates in neutrophil recruitment and activation, which in turn can induce complement activation in AAV (38). C5a induces tissue factor expression by neutrophils, leading to factor X activation and thrombin generation, which in turn cleaves C5 into C5a in APS (32).

Ultimately, several triggers of complement activation and effectors may contribute to cell and tissue damage in heterogeneous human diseases. The identification of specific triggers of complement activation and fine pathophysiological mechanisms resulting in cell and tissue complement-mediated injury is needed to determine the best therapeutic target within the cascade. Complement inhibitor anti-C5 monoclonal antibody (eculizumab, and more recently its long-acting form, ravulizumab) is the gold standard in two complementopathies, aHUS and PNH, and has obtained Food and Drug Administration (FDA) approval for MG and NMOSD. Avacopan is a C5aR1 antagonist that has also been approved by the FDA for patients with AAV, another disease with a major complement contribution. Understanding the detailed mechanisms of complement activation and complement-mediated damage is necessary to guide the prescription of new complement inhibitors.

## Overview of the Tests Exploring Complement Activation

### Quantification of Complement Components

Currently available tests mainly consist in quantification of individual complement components or activation products.

- For the quantification of individual complement proteins in plasma, various types of immunoassays are used to determine the concentration of individual complement components. The most common is nephelometry. Polyclonal antibodies to component are added in excess of the sample and bind to their target. Quantification is performed by passing a light beam through the sample, which is distorted by the IC that have formed (44).
- Quantification of complement activation products corresponding to cleavage fragments or complement protein complexes (C3a, C3dg, C4a, C4d, Ba, Bb, C5a, C3bBbP, MASP2, and sC5b-9) is possible. Several assays have been described, mostly based on the recognition of a neoepitope of the complement component in an enzyme-linked immunosorbent assay (ELISA) format. Thus, C4a and C4d reflect CP/LP activation, Ba, Bb, and C3bBbP reflect AP activation, MASP2 is a key enzyme in LP activation (45) and increasing soluble C5b-9 reflects TP activation (46). C3a and C5a are common to the three activation pathways.
- Detection of auto-Abs (anti-FH, FB, C3b, C3bBb, and C1q) targeting complement proteins can be performed using ELISA (14).

### Functional Assays

• Quantification of complement function is used to explore the activity of a pathway or the entire cascade.
- In hemolytic assays, CP activation can be assessed by incubating patient sera with sheep erythrocytes coated with rabbit anti-sheep red blood cell antibodies (47). In this assay, termed the CH50 assay, C1q binds to immunoglobulins, initiates the formation of CP C3 convertase, and leads to MAC assembly and erythrocyte lysis. Hemoglobin release is determined to calculate the number of hemolytic sites per cell. Activation through AP can be assessed using rabbit or guinea pig erythrocytes, which are activators of human AP, incubated with patient serum added to ethylene glycol-bis(β-aminoethyl ether) (EGTA), which chelates Ca2+ and inhibits activation *via* CP and LP (48). This hemolytic assay is termed the AP50 assay.
- Liposomes coated with an activator can be used in a similar manner to CH50 assays (49). The main difference is the readout, which consists of the unquenching of a fluorescent dye and not the lysis of erythrocytes.
- Assays based on ELISA method can also be used to explore the function of the three pathways. Microtiter plate wells are coated with recognition structures specific to each pathway (IgM for CP, mannan or acetylated bovine serum albumin for LP, and LPS for AP). Patient serum is added and incubated under conditions in which only one pathway is operative at any given time; the other two pathways are blocked. Finally, activation capacity is detected through the formation of the C5b-9 complex by monoclonal antibodies targeting a neo-epitope in complex-bound C9 (50).
• Different hemolytic assays have been developed to explore specific steps of the AP.
- Sanchez-Corral et al. (51) developed a hemolytic assay to study FH functional defects in aHUS. The assay relies on the knowledge that sheep erythrocytes are highly sialylated and favor FH binding, whereas their membrane complement regulators are incompatible with human complement proteins. Therefore, they are protected from complement lysis due to the binding of human FH to their surface. In the assay, sheep erythrocytes are incubated with human plasma in Mg-EGTA buffer, allowing activation of AP only. Normal plasma does not induce lysis, whereas aHUS plasma with FH functional defects (mutations or autoantibodies) induces lysis under these conditions (51).
- Hemolytic assays can also be used to study the stabilization of cell-bound AP convertases (52). This assay has been used to detect C3Nef in C3G cells. Sheep erythrocytes bearing C3 convertase C3bBb (generated by exposure of sheep erythrocytes bearing C3b to FB and FD) were incubated with patient IgG. C3Nef activity correlates with residual C3bBb hemolytic sites, and lysis is developed by the addition of rat serum.
• Staining of tissue sections for the deposition of complement activation products can provide information about local complement activation in tissue. This can be performed by immunohistochemistry or immunofluorescence (53). For example, this technique has been used to study C5b-9 deposition in the skin of patients with aHUS (54).

**Figure 2:**
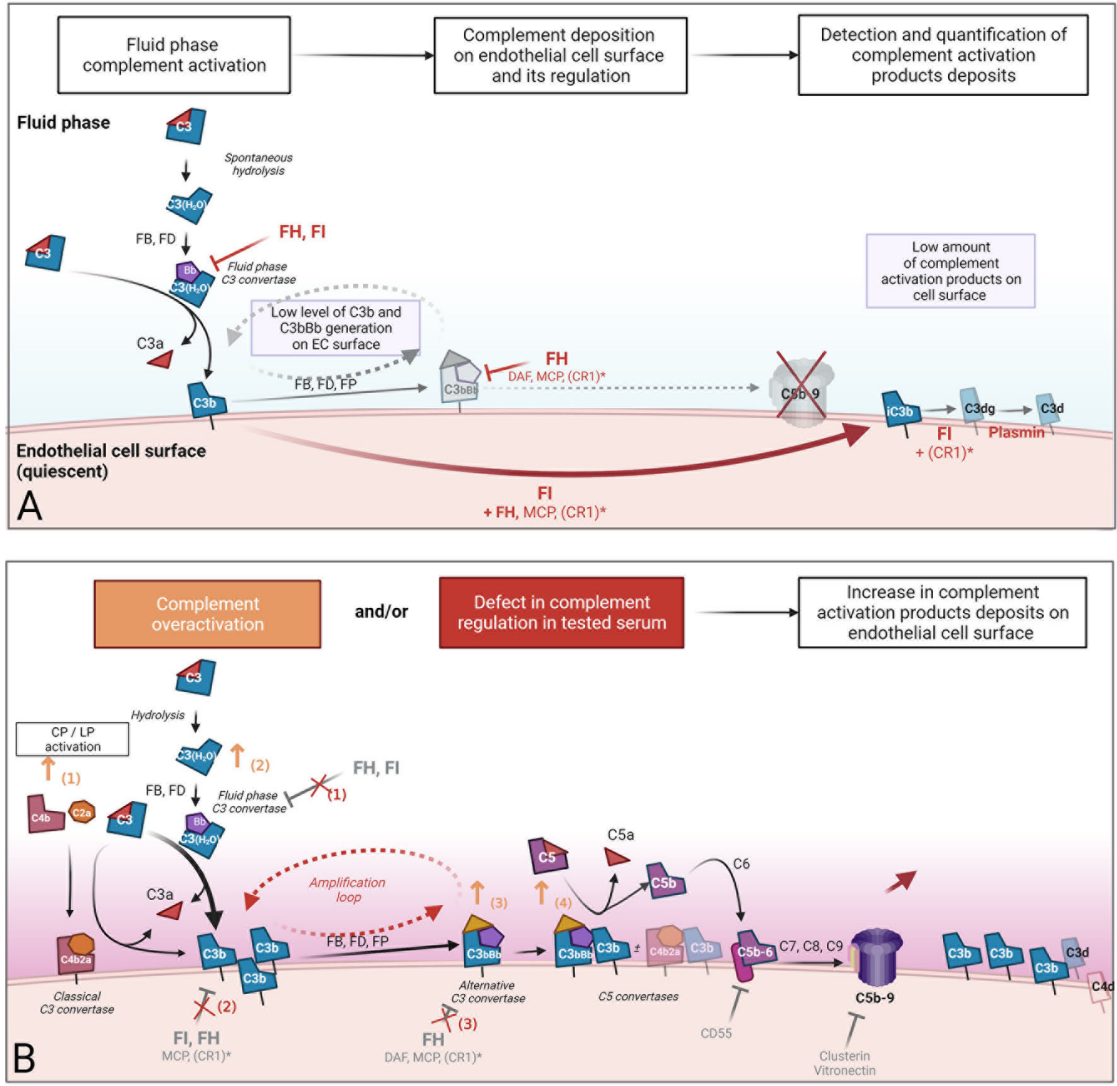
Concept of *ex vivo* complement deposition on endothelial cells. An *ex vivo* endothelial assay was developed to reproduce human pathological conditions and their complexity. The assay consists of the detection and quantification of complement component deposition on the cultured endothelial cells (EC) surface after incubation with human serum. The EC surface was used as the regulatory surface. **(A)** In serum from healthy individuals, the alternative pathway is active at low levels but tightly regulated in the fluid phase by regulators, resulting in a very low level of complement activation product deposition on the EC surface. The detection of an increased complement deposition when incubation is performed with pathological serum **(B)** could result in either i) complement overactivation that overwhelms EC capacity of regulation (orange) or ii) defect in complement regulation in fluid or solid phase. Both are induced by tested human serum incubated with EC. Orange arrows represent some mechanisms involved in complement overactivation in serum (1): the participation of a coactivation of classical/alternative pathway due to pathological immunoglobins, immune complexes, or lectin pathway activation by polymeric IgA in IgA nephropathy (2), an increase in the formation of fluid phase C3 convertases in the presence of heme or fluid phase activating surface, and the stabilization of C3 (3) or C5 (4) convertases by pathological immunoglobulins, such as C3 and C5 nephritic factors. Red crosses represent potential defects in alternative complement pathway regulation in the fluid phase (1) and on the cell surface (2, 3). These defects in complement regulation could be the consequence of inhibition of the main alternative pathway regulator FH due to anti-factor H antibodies (such as in aHUS), a lack of function, or a quantitative deficiency of FH and FI due to pathological genetic variants. *CR1: weak expression of CR1 on endothelial cells. CR1, complement receptor 1 (CD35); FB, factor B; FD, factor D; FH, factor H; FI, factor I; FP, properdin; MCP, membrane cofactor protein.

These tests allow only the characterization of a specific molecule or step of the complement cascade. To reproduce human pathological conditions and their complexity, several authors have proposed the use of an *ex vivo* endothelial assay. The assay detects and quantifies complement component deposition on the EC surface after incubation with human serum. The EC surface is used as the regulating surface. The objective is to detect abnormal complement deposition that could result from either complement overactivation exceeding the capacity of regulation, or from a defect in complement regulation in fluid or on the EC surface. The *ex vivo* endothelial assay is presented in **Figure 2**.

We next discuss the advantages and limits of this functional approach.

## Study of Complement Deposition on Cultured Endothelial Cells

### Heterogeneity of Endothelial Cells Populations and Their Complement Regulation

EC line blood vessels and constitute an active regulatory organ that has been implicated in vascular homeostasis, permeability regulation, vasomotor tone, angiogenesis, and diapedesis of immune cells (55). As first barrier between the blood and interstitium it is in constant equilibrium with the environment. Thus, heterogeneity in the structure and function of EC is a core property of the endothelium, allowing diverse vascular functions and regional specificity (56, 57). This diversity can be partially explained by a distinct transcriptional profile (58) in relation to neighboring cells (59). Hence, EC from different blood vessels have distinct and dynamic expression profiles of complement components and regulators, which may explain the different susceptibility and specific organ tropism observed in some complement-mediated diseases (60).

At a steady state, EC can produce most complement components and express high levels of complement regulators on their membranes (**Table S1**). Under inflammatory conditions, complement component production and regulatory protein expression are modified (**Table 1**). In addition to the steady state, the modulation of complement protein expression under inflammatory conditions differs according to the EC type and probably contributes to a specific damage mediated by AP and the different organ tropisms observed in complement-mediated diseases. Sartain et al. demonstrated that resting or tumor necrosis factor (TNF)-stimulated brain microvascular EC expressed higher levels of regulatory molecules (FH, FI, CD46, CD55, and THBD), generated lower levels of C3a and C4a, and enhanced lower degree AP activation (measured by lower Ba generation) than human renal glomerular EC (HRGEC) (61). The authors also demonstrated a slight increase in CD46 expression, decrease in thrombomoduline (TM), and increase in C3 and FB transcription in HRGEC exposed to TNF (62). These results agree with the prior demonstrations of an increase in C3 and FB production by human umbilical vein EC (HUVEC) exposed to TNF (63), increased FH transcription and production by HUVEC exposed to interferon (INF) gamma (64), increased C2, FH, FB, and C1inh transcription, and decreased C3 production by HUVEC exposed to INF gamma (65). May et al. compared the properties of four EC types (HRGEC, glomerular EC (GEnC), human microvascular EC (HMEC), and HUVEC) in the resting state and after overnight exposure to heme (66). While there was no difference in expression of regulatory factors (MCP, CD55, TM) at resting state, after overnight heme exposure, C3 deposits on glomerular EC were greater than on other EC. This was associated with, and possibly explained by, weaker FH binding and TM upregulation and lower upregulation of heme-oxygenase 1 (cytoprotective heme-degrading enzyme) compared to HUVEC. Moreover, HUVEC, but not EC, of glomerular origin were protected from complement deposition after re-challenge with heme (**Table S2**).

**Table 1:** Production of distinct complement components and expression of regulators according to endothelial cell type after stimulation.

		TNF	INF gamma	IL1 beta	Heme
**HRGEC**	C3	↑	↑	→*	
** **	C4	→*	↑	→*	
** **	C5	→*		→*	
** **	FB	↑		→*	
** **	FD	→*		→*	
** **	Properdin	↓*		→*	
** **	FH	→		→*	
** **	FI			→*	
** **	TM	↓		↑	
** **	CD46	↑		→	↓
** **	CD55	→		→	↓
** **	CD59	→		→	
** **	E-selectine	↑	→		
** **	C3aR	↑			
** **	C5aR				
**BMVEC**	C3	↑			
** **	C4	→*			
** **	C5	→*			
** **	FB	↑			
** **	FD	→*			
** **	Properdin	↓*			
** **	FH	→			
** **	CD46	↑			
** **	CD55	→			
** **	C3aR	↑			
** **	C5aR				
**HMEC**	E-selectine	↑	→		
** **	C3		→		
** **	C4		↑		
** **	CD46				↓
** **	CD55				→
**HUVEC**	C2		↑		
** **	C3	↑	→/↓	↑	
** **	C4			→*	
** **	C5			→*	
** **	FB	↑	↑	↑*	
** **	FD			→*	
** **	Properdin			→*	
** **	FH		↑	↓	
** **	FI			→*	
** **	TM	↓		→	
** **	CD46	↑		→	↓
** **	CD55	↑		↑	↓
** **	CD59	→		→	→
** **	E-selectin	↑	→		
** **	P-selectin				↑
** **	C1-inh		↑		

The data presented here are mainly concerned with protein expression.

↑, increase in protein production; ↓, decrease and →, no change.

*Denotes transcriptomic data. For details, please refer to **Table S2**. BMVEC, brain microvascular endothelial cells; HMEC, human microvascular endothelial cells; HRGEC, human renal glomerular endothelial cells; HUVEC, human umbilical vein endothelial cells.

EC used for *ex vivo* experiments comprise two types: conditionally immortalized EC (CI-EC) and primary EC (**Table 2**). Primary EC can be difficult to isolate and maintain in culture, and have a limited lifespan. Moreover, differences in the genetic background of individual donors can lead to interexperimental variability. In particular, inter-individual heterogeneity in complement regulator expression at the EC surface cannot be excluded. CI-EC has been developed to overcome these difficulties. HMEC-1 and CI-GEnC are HMEC and GEnCs, respectively, that have been transfected with SV40 large T antigen (67, 68). EA.hy926 cells were obtained by fusing HUVEC with A549 cells obtained from human lung carcinoma (69). The EA.hy926 cells were used to generate glycosylphosphatidylinositol-anchored complement regulatory protein-deficient cells when treated with phosphatidylinositol-specific phospholipase C. These cells have been used along with the PIGA-mutant TF-1 to study complement deposits by confocal microscopy and flow cytometry after incubation with serum from patients with thrombotic microangiopathy (TMA), this test was called the modified Ham test (70). After incubation with serum from aHUS patients, cell surface C5b-9 deposits were reportedly higher than after incubation with thrombotic thrombocytopenic purpura (TTP) serum. Therefore, this test has been considered a tool to distinguish aHUS from TTP. It is important to note that sC5b-9, reflecting terminal pathway activation and regulation, is elevated under both aHUS and TTP plasma conditions (71, 72). One possibility is that both conditions are associated with complement activation. However, in aHUS, complement overactivation exceeds alternative and terminal pathway regulation, leading to C5b-9 deposits. In contrast, in TTP, complement activation is counterbalanced by complement regulation, leading to sC5b-9 release, but not C5b-9 deposits in the modified Ham test.

**Table 2:** Endothelial cells used for *ex vivo* experiments.

	Conditionally immortalized	Primary
Macrovascular		HUVEC
Microvascular	CI-GEnC HMEC-1	BMVEC HRGEC BOEC

BMVEC, brain microvascular endothelial cells; BOEC, blood outgrowth endothelial cells; CI-GEnC, conditionally immortalized human glomerular endothelial cells; HMEC, human microvascular endothelial cells; HRGEC, human renal glomerular endothelial cells; HUVEC, human umbilical vein endothelial cells.

Micro- or macrovascular origin of the EC tissue lineages also needs to be considered. Complement-mediated EC injury demonstrates specific cell tropism according to pathophysiological processes. In HUS and TTP, microvascular EC of dermal, renal, and cerebral origin are more sensitive to apoptosis, whereas microvascular EC of pulmonary and hepatic origin and macrovascular EC are resistant (73). Distinct sensitivity of EC to complement attack has also been explored in aHUS and heme exposure. The demonstration of a distinct EC response in terms of complement regulator expression after a trigger (here heme) was proposed to partially explain the kidney tropism in this disease (66).

HUVEC are primary macrovascular EC isolated from human umbilical cords. These are the most frequently used cells for *ex vivo* assays (74). If tissue specificity is required, HRGEC (75) or GEnCs (76), which are both isolated from human glomeruli, can be used. More recently, the use of blood outgrowth EC obtained from the differentiation of circulating marrow-derived endothelial progenitor cells isolated from peripheral blood has been proposed (77).

### Comparative Analysis of the Available Endothelial Assays

These tests consist of the quantification of complement activation products (C3 activation fragments and C5b-9) deposits on EC by immunofluorescence (IF) measured by confocal microscopy or flow cytometry (fluorescence-activated cell sorting, FACS) after incubation with a serum sample of interest. Different protocols have been proposed to study complement activation on the EC surface in several pathological conditions, including aHUS (30, 78–88), TMA of other etiologies (89, 90), HELLP syndrome and pre-eclampsia (91), C3G (14, 92), lupus nephritis (LN) (93, 94), APS (95, 96), SCD (29), hemolytic anemia (97) and hyperhemolytic transfusion reaction without hemoglobinopathy (98).

**Figure 3:**
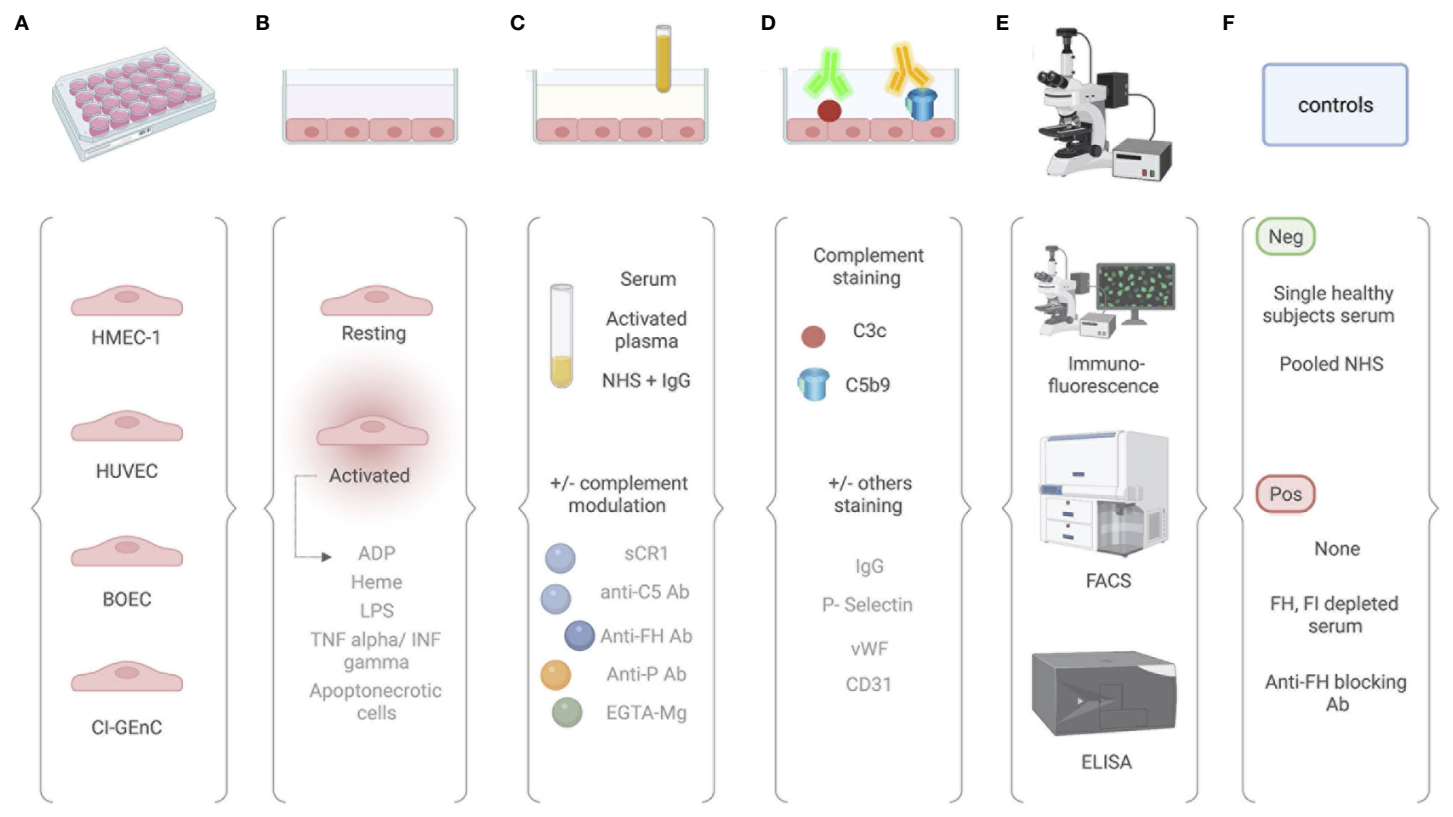
Comparative analysis of different protocols used for the *ex vivo* complement activation test with endothelial cells. **(A)** The *ex vivo* test for measuring complement attack on endothelial cells can be performed on different endothelial cells, including human dermal microvascular endothelial cells (HMEC-1), human umbilical vein endothelial cells (HUVEC), blood outgrowth endothelial cells (BOEC), and glomerular endothelial cells (GEnC). **(B)** Cultured EC are then used at their resting state or after an activation by either ADP, heme, LPS, TNF/INF gamma, or apoptonecrotic cells. **(C)** EC are incubated with sample of interest. Either serum or activated plasma (consisting of patient citrated plasma mixed 1:1 with control serum pool) or normal human serum with addition of the protein of interest (e.g., IgG). Complement activation can be modulated in by addition of sCR1, anti-C5 antibody, anti-FH antibody, anti-properdin antibody, or EGTA-Mg buffer. **(D)** Complement activation products are then revealed by fluorescent tagged antibody. Antibody directed again C3c or C5b9 can be used. According to the context, staining for other molecules have been proposed and include IgG, P-selectin, vWF, and CD31. **(E)** Quantification is then performed using immunofluorescence scanning, flow cytometry, or ELISA. **(F)** Controls are required and vary according to the protocols.

The general procedure of the *ex vivo* assay and the different protocols are presented in **Figure 3**.

#### To Pre-Activate or Not Pre-Activate EC?

Resting EC or EC pre-activated by cytokines, ADP, or heme can be used (**Table S3**) to provide additional information.

When resting HUVEC were incubated with aHUS FB mutants added to FB-depleted normal human serum (NHS), enhanced C3b/iC3b-fragment deposition as measured by an anti-C3c-reacting antibody was observed (78). The same result was obtained for some cases when aHUS patient serum was incubated with resting HUVEC (30, 79). Nevertheless, incubation with NHS depleted in FB and reconstituted with other aHUS FB variants (80) or incubation with aHUS serum from patients carrying some C3 or FH variants (30, 79) may be insufficient to induce C3 or C5b-9 deposits. When quiescent HMEC-1 were incubated with aHUS serum from patients carrying mutations in FH, FI, C3, or FH/CFHR1 hybrid, enhanced C3c or C5b-9 deposition was reported only if serum was collected during the acute phases of the disease and not after reaching remission (82, 89). Furthermore, deposits on quiescent HMEC-1 are better correlated with relapse risk during the tapering or discontinuation of eculizumab (85).

To increase test sensitivity, the authors proposed pre-activating EC. Modifications in surface-bound protein expression enable complement activation. This was achieved in the case of P-selectin expression on HMEC-1 pre-activated with ADP, LPS, or thrombin (82) or P-selectin expression on HUVEC or GEnC pre-activated with heme (30, 80, 99), which could allow C3b binding and C3 convertase formation. Enhanced formation of C3 fragments by TNF/IFN pre-activation and C5b-9 deposition by ADP pre-activation on HUVEC or HMEC-1 cells was described after incubation of these cells with serum from asymptomatic carriers of mutations in AP regulatory proteins or C3 (79, 82). The normal range was established when pre-activated EC were incubated with sera from healthy donors. In addition, serum from healthy family members without the mutation was within the normal range in this assay (79).

#### What Kind of Blood Samples Might Be Incubated With EC?

Serum has been used as the source of complement proteins in the vast majority of the tests described above. One limitation of these tests, particularly when deposits are detected by IF, is the variation in the results, reportedly from 30% to 52% when activated HMEC-1 were incubated with serum collected at the acute phase of aHUS (91). To reduce this variation, Palomo et al. proposed the use of activated plasma, which refers to citrated plasma mixed 1:1 with a control serum pool. Using this approach, the authors derived a coefficient of variation of 9% to 18% (91). C3 consumption by the patient or loss of C3 activity during the pre-analytical phase are also potential factors responsible for this variation (4). Finally, for all complement assays and to avoid *in vitro* complement activation, proper blood collection and processing must be achieved (100). Processing of plasma or serum sample must be performed within a few hours of collection, with storage at –80°C and defrosting immediately before use to avoid repeated freezing and thawing.

To explore the functional consequences of autoantibodies against C3 and properdin in SLE, Vasilev et al. and Radanova et al. incubated HUVEC with NHS supplemented with purified IgG from patients positive for such autoantibodies (93, 94). Using this strategy, complement deposition on EC can be directly ascribed to the addition of autoantibodies to NHS. The same approach was applied for anti-C3b/FB autoantibodies in patients with C3G (14). To understand the mechanism behind complement deposits on EC from patients with SCD, microvesicles from normal or patient-derived erythrocytes were added to normal serum to model the disease condition. Enhanced binding of the C3 activation products was demonstrated (29, 101).

#### Which Controls Are Relevant?

Most often, NHS is used as a negative control (14, 29, 30, 78–83) (**Table S4**). An important aspect to consider is the inter-individual variability in deposits induced by normal sera. FACS analysis has revealed that this variability was relatively low when sera from 50 healthy donors were tested (79). However, this is a concern, particularly when deposits are detected by IF. This has not been directly reported, but has been suggested by the use of pooled sera in more recent papers (85, 91) and our own experience. Aiello et al. reported that C3 and C5b-9 deposits obtained after a single healthy subject serum (N=12) incubation range from 0.5 to 1.5 fold increase of stained surface area compared to pooled serum (from 10 healthy donors) run in parallel (87).

Several authors did not use any positive controls for their experiments (82, 83, 85, 90, 91). The comparison was only made with the deposits obtained with negative controls. It might be interesting to position the results on a scale. Positive controls with published data are FH or FI depleted NHS (14, 79, 81) or normal serum supplemented with blocking anti-FH antibodies targeting the N-terminus or C-terminus (30, 80) or with FH19-20, corresponding to the two last domains of FH, able to compete with the full FH protein for cell surface binding (97).

The main issues with this type of assay are the lack of validated international standards as well as standardized positive and negative controls. The variability of the results in samples from healthy donors needs to be studied extensively to determine the appropriate cutoff. In addition, the impact of C3 or other complement protein consumption in the patient and the influence of the pre-analytical phase must be determined to avoid false positive and false negative results.

#### Which Deposits Should Be Measured?

The objective of these tests is to demonstrate and explore complement overactivation or dysregulation on the EC surface after incubation with blood samples of interest. This is enabled by quantification of the deposition of complement component products resulting from activation or regulation. C3c (a common epitope to C3, C3(H2O), C3b, and iC3b) (which reflects C3 convertase activity and the early phase of the complement cascade) can be detected by polyclonal anti-C3c antibody. Antibody targeting C5b-9 reveals the final step of the cascade. When a signal is detected on the cell membrane, it can be assumed that the detected fragment is C3b or iC3b covalently attached to the surface. Nevertheless, heme-activated EC and likely ADP-activated EC (102, 103) express P-selectin, which recruits C3b, C3(H2O), and a C3(H2O)-like form of C3 generated after contact with heme (30, 99). Properdin also binds to heme-exposed or stressed EC, promoting complement activation in a similar manner without covalent C3b binding (97). This is an additional mechanism for amplification of complement activation on the EC surface. C5b-9 deposits may be more relevant in identifying dysregulation at any step. Nevertheless, early dysregulation can induce C3 activation fragment deposits without C5b-9 formation because of TP regulation. C5b-9 is readily detectable by IF but is much more difficult to detect by FACS because of the weak shifts of the peaks. To test for CP participation, the presence of C4d-positive deposits was also investigated (82, 89). Staining can also be performed under the same conditions for von Willebrand Factor, C5aR1, P-selectin, and others (87).

#### Evaluation of Activated Pathways

The test can be modified to assess which complement pathway is activated in given pathological settings. The test can be performed under different conditions to avoid CP and LP contributions, which include C2 (30) or C1q (104) depleted NHS, addition of SCR1 (87) or Mg-EGTA buffer (30, 78, 93, 94). EGTA chelates Ca2+, which is crucial for CP and LP activation, whereas AP depends on Mg2+. If AP has to be inhibited, FB-depleted NHS can be used. These reagents are applicable for test conditions, where the activating factor is added externally to the serum (i.e., IgG, heme, microvesicles, etc.). When patient samples are used directly, the same effect can be achieved by inhibiting C1q, C4, FB, or properdin with blocking antibodies, protein constructs, or small molecules, if available (97). Quantification of complement activation products (split fragments generated by cleavage of complement components or protein complexes when activated components bind their target (i.e., C3a, C4a, Ba, Bb, C5a, and sC5b-9) in the supernatant might be an additional element to study complement cascade activation.

#### Which Techniques Are Used for Detection and Quantification?

The two main detection techniques commonly used are FACS and IF. HUVEC pre-activated with heme and then incubated with NHS or aHUS serum showed results similar by FACS or IF detection (30). IF directly analyzes deposits on EC grown on slides. FACS requires a cell detachment step before staining, with the potential risk of losing a part of the deposit signal. In contrast, as mentioned by Gavriilaki et al., obtaining quantitative data by IF requires confocal microscopy and further analysis using specialized software (70). When IF is used, the area occupied by fluorescent staining in fields systematically digitized along the surface is quantified. The quantified results expressed as the mean of the square number of pixels per field are compared with the negative control (82, 83, 85, 89, 91). Considering the number of EC on which fluorescence has been measured and the staining intensity might appear relevant. In contrast, FACS allows the rapid and objective quantification of deposits.

#### What Are the Functional Consequences of Such Deposits?

If enhancement of complement fragment deposits on EC is interpreted as pathogenic, the functional consequences of such deposits must be questioned. Lactate dehydrogenase release from EC reflects cell damage. This release can be measured in the cell culture supernatant (104). Analysis of complement deposits can also be associated with a cell viability assay, corresponding to a colorimetric assay based on cleavage of the WST-1 tetrazolium salt by mitochondrial dehydrogenases in viable cells (70). Cellular integrity can be verified by May-Grunwald Giemsa staining (88). Direct cell death rarely occurs under these experimental conditions. Experiments testing cell activation status by complement overactivation have not been reported in the literature and are needed to further understand the impact of complement on endothelial injury. Analysis of transcriptomic modifications in EC exposed to complement deposits under several conditions could also be of interest.

### Clinical and Therapeutic Relevance of the Obtained Results

The *ex vivo* EC assay, consisting in the quantification of complement activation products (C3 activation fragments or C5b-9) deposits on EC (by IF measured on confocal microscopy or FACS), after incubation with a serum sample of interest, was first used for specific characterization of complement component abnormalities (78–81, 83) or exploration of mechanisms implicated in EC injury (30) in the main complementopathy, aHUS. The assay was then used to demonstrate and explore complement activation and participation in the pathophysiology of several diseases, including C3G (14, 92), HELLP syndrome and pre-eclampsia (91, 105), TMA associated with severe hypertension (89, 106), drug-induced TMA (90), SCD (29), hemolytic anemia (97, 98), SLE (93, 94), and APS (96). Demonstration of increasing complement deposits on EC incubated with pathological sera is not sufficient to determine what is responsible for complement activation at the EC surface. Modulation of the test conditions can help in detailing complement activation. This was the case when complement activation was inhibited by the addition of hemopexin to the sera of patients with SCD (29).

Noris et al. and Galbusera et al. also proposed the use of this *ex vivo* EC test to monitor eculizumab therapy in patients with aHUS (82, 85). During eculizumab tapering or discontinuation, disease relapse preceded or was associated with an increase in C5b-9 deposits on resting HMEC-1 in all patients. In contrast, only one patient without relapse showed increased deposits (85). In clinical practice, CH50 is the only routine test used to monitor eculizumab therapy. CH50 is reportedly strongly suppressed in patients receiving eculizumab according to the standard protocol. However, CH50 does not allow monitoring of eculizumab dosage tapering or discontinuation, as it is not well correlated with relapse risk (82, 85). Eculizumab therapy monitoring using the Wieslab^®^ complement system screen (107) or the modified Ham test (108) has also been proposed. Thus, the *ex vivo* EC assay could represent a test of interest to a better personalized complement-blocking therapy, but first needs to be more standardized.

This test can also be used to better classify and assess the prognosis of specific diseases. This is the case for hypertensive TMA, as Timmermans et al. demonstrated in a cohort of hypertension emergencies associated with TMA (106). The authors demonstrated a statistical association between increased C5b-9 deposition in the EC *ex vivo* test and kidney survival. Moreover, they reported an improvement in renal function for those with increased deposits treated with eculizumab. The authors proposed a classification of TMA-hypertensive emergency based on the EC *ex vivo* test (106).

Finally, many new anti-complement drugs targeting specific steps of the cascade have been under development in recent years (109). A standardized and validated assay to study complement activation could be a useful tool in their development.

## Discussion and Conclusion

The increasing demonstration of complement involvement in the pathophysiology of many human diseases has mandated the development of tools to finely explore complement activation. Complement is a complex enzymatic cascade that is highly regulated in constant interplay with its environment. The current arsenal for complement exploration does not provide functional characterization and does not report on the complex interplay between complement and its environment, particularly the cell surface.

The development of tests with these capabilities could allow for a deeper exploration of the mechanisms of complement activation in several diseases. This information could inform the development of a complement blocking therapeutic strategy based on pathophysiological mechanisms.

**Figure 4:**
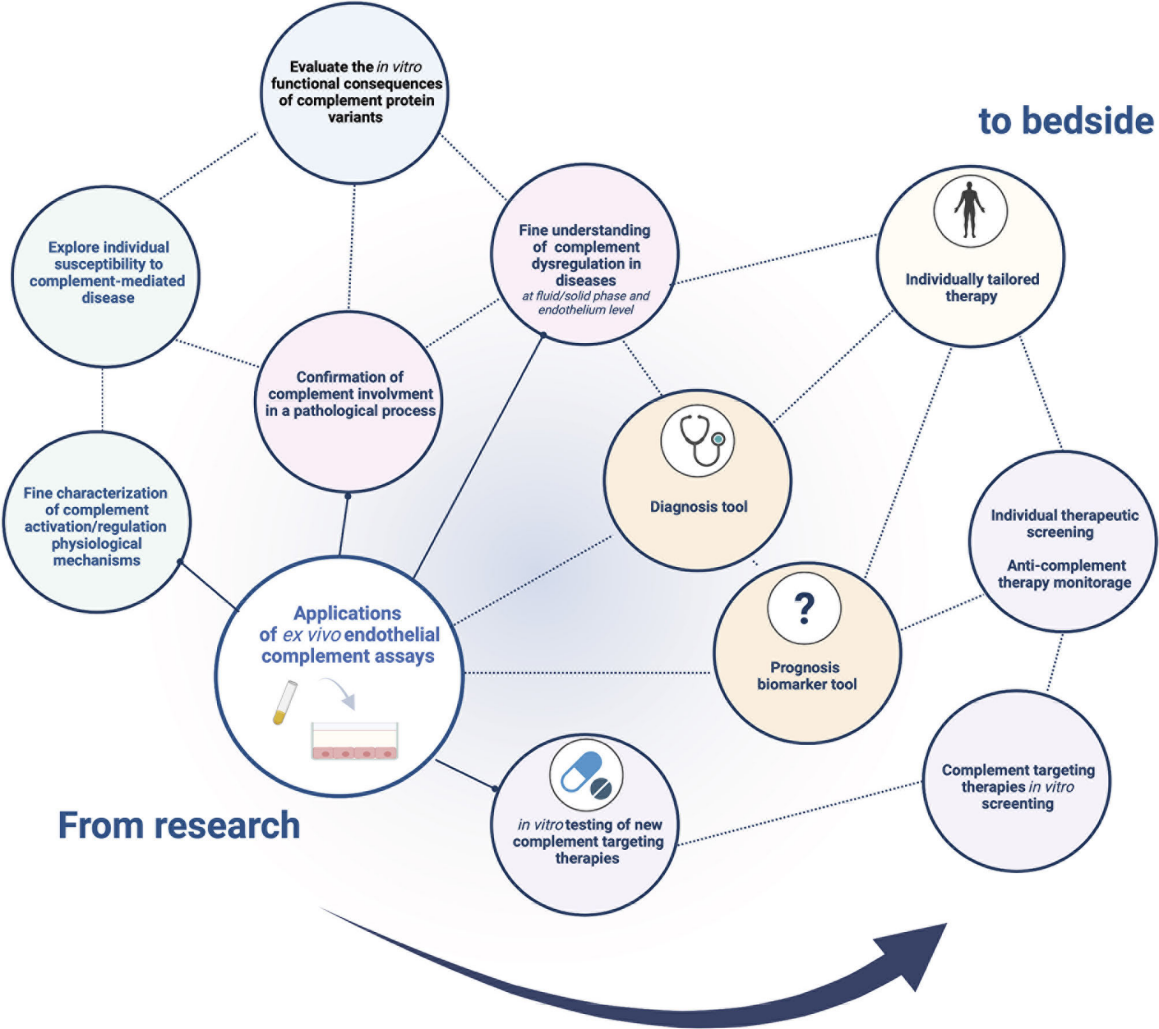
Current and future application fields of the *ex vivo* complement activation test on endothelial cells. There is a wide range of potential applications of *ex vivo* complement activation tests in endothelial cells. Currently used to decipher *in vitro* complement pathophysiology in research, a standardized test would represent a promising tool in clinical and therapeutic fields, paving the way for tailored medicine in complementopathies.

*Ex vivo* complement activation on EC represents a promising tool for demonstrating and exploring complement activation (**Figure 4**). It not only recapitulates complex complement cascade regulation *in vivo*, but also allows modification of several steps of the experimental procedure to characterize complement activation mechanisms.

However, there are still unanswered questions hindering broad used. The first is the variability in the results and the inter-individual variability in deposits induced by normal sera. Comprehension of the precise mechanism responsible for complement deposition in this assay would improve its better use. The second issue is to standardize the main steps of the procedure to improve the interexperimental comparison.

The use of such a test could be multiple, including molecular functional characterization, disease pathophysiology exploration, prognosis classification, complement targeting drug development, and complement therapeutic monitoring. The use of standardized conditions will expand the field of this promising tool.

## Author Contributions

M-SM, SC, and LR conceptualized and conceived the manuscript. M-SM drafted the manuscript, including the literature search, reading, and writing. SC, VF-B, AD, and LR edited and critically evaluated the manuscript. All authors have contributed to the manuscript and approved the submitted version.

## Funding

This research was funded by ANR JCJC 2020 COMSIGN.

## Conflict of Interest

The authors declare that the research was conducted in the absence of any commercial or financial relationships that could be construed as a potential conflict of interest.

## Publisher’s Note

All claims expressed in this article are solely those of the authors and do not necessarily represent those of their affiliated organizations, or those of the publisher, the editors and the reviewers. Any product that may be evaluated in this article, or claim that may be made by its manufacturer, is not guaranteed or endorsed by the publisher.
