# Supplementary material for: Ex Vivo Test for Measuring Complement Attack on Endothelial Cells: From Research to Bedside
Source: Front Immunol. 2022 Apr 12;13:860689. doi: 10.3389/fimmu.2022.860689 (PMC9041553; doi:10.3389/fimmu.2022.860689)
Supplement: Supplementary file 1 [file Table_1.docx]

**Table S1**: Characteristics of the endothelial cells used for in vitro experimentation at resting state

| Characteristics | Endothelial cells |
| --- | --- |
| Surface proteins expressed at resting state | |
| DAF (CD55) | HMEC1≈HUVEC≈CI-GEnC≈HRGEC (FACS) (1); HRGECx2,5 > HUVEC (FACS) (2); BMVEC x2 > HRGEC (FACS) (3) |
| CD59 | HRGEC x1,4 > HUVEC (FACS) (2) |
| MCP (CD46) | HMEC1≈HUVEC≈CI-GEnC≈HRGEC (FACS) (1), HRGEC x1,5> HUVEC (FACS) (2); BVMEC ≈ HRGEC (FACS) (3) |
| CR1 (CD35) | No/very low on HUVEC and CI-GEnC (FACS) (4) |
| Thrombomodulin | HMEC1≈HUVEC≈CI-GEnC≈HRGEC (FACS) (1); HRGEC x1,3 > HUVEC (FACS) (2) Positive on BOEC (FACS) (5) |
| C3aR / C5aR | BMVEC x2 > HRGEC / Undetectable (FACS) (3) |
| PECAM-1 (CD31) | HUVEC>HRGEC (IHC) (6) HMEC≈HUVEC>HRGEC (ELISA Cell) (7); CI-GEnC≈HRGEC (IF, WB)(8) HMEC1≈HMEC (FACS)(9), Positive on BOEC (IF) (5) (FACS) (10) |
| ICAM 2 | CI-GENC≈HRGEC (WB) (8) |
| VEGFR2=flk1 | HUVEC≈HRGEC (IHC) (6) , CI-GEnC≈HRGEC (WB) (8) + on BOEC(IF) (5) |
| VE-Cadherin | CI-GEnC≈HRGEC (IF, WB) ((8) + on BOEC (FACS) (L) ((IF) (5) |
| tPA (supernatant) | HUVEC>HRGEC (ELISA) (11) |
| uPA(supernatant) | HUVEC<<HRGEC (ELISA) (11) |
| PAI-1 (supernatant) | HUVEC>>HRGEC (ELISA) (11) |
| Ratio tPA+uPA:PAI1 | HUVEC antifibrinolytique (1:55) vs HRGEC profibrinolytique (24:1)(11) |
| VCAM-1 | HMEC, HUVEC, HRGEC: Fine granular pattern on the surface (IF) (7) |
| VWF | HUVEC>HRGEC (IHC) (6) ; CI-GEnC≈HRGEC (IF,WB) (8); HMEC1≈HMEC (IF) (9) ; Positive on BOEC (IF) (5) |
| Genes expression at resting state | |
| CD46 / CD55 / CD59 | HRGEC> HUVEC : x0,3/x0,3/x1,7 (RTqPCR) (2); BOEC≈HUVEC (RTqPCR)(10); CD46 et CD55: BMVEC x 6 / x 14 / NA > HRGEC (RTqPCR) (3) |
| Properdin / FD / CFH | HRGEC x7/ x3/ x3 >HUVEC (RTqPCR) (2); BMVEC x 4 < / x 4 < / x3,5 > HRGEC (RTqPCR) (3) |
| C3 / C5 / CFB / CFI / C4 / VWF / ADAMTS13 | HUVEC≈HRGEC (RTqPCR) (2); BMVEC x14 / x3 / x3/ x18 / x5 / NA / NA > HRGEC (RTqPCR) (3) |
| Thrombomodulin | HRGEC x2 > HUVEC (RTqPCR) (2); BMVEC x2,5 > HRGEC (RTqPCR) (3) |
| FI | BMVEC x18 > HRGEC (RTqPCR) (3) |
| C3aR / C5aR | BMVECx 3,8 < HRGEC / Undetectable (RTqPCR) (3) |
| VE-Cadherin | HRGEC<HUVEC (RTqPCR) (12) |
| PAI-1 / Thrombospondin / Fibronectin | HRGEC≈CI-GEnC (RTqPCR) (8) |
| Specific EC genes expression at resting state | |
| PECAM-1 | CI-GEnC≈HRGEC (RTqPCR) (8) |
| ICAM2 | CI-GEnC≈HRGEC (RTqPCR) (8) |
| VEFGR2 | CI-GEnC>HRGEC (RTqPCR) (8) |
| vWF | CI-GEnC>HRGEC (RTqPCR) (8) ; BOEC≈HUVEC (RTqPCR) (10) |

Abbreviations:

BMVEC: Brain Microvascular Endothelial Cells

BOEC: Blood outgrowth endothelial cells

CI-GEnC: Conditionnally Immortalized Human Glomerular Endothelial Cell

HMEC: Human Microvascular Endothelial Cells

HRGEC: Human Renal Glomerular Endothelial Cell

HUVEC: Human Umbilical Vein Endothelial Cells

**References**

1. May O, Merle NS, Grunenwald A, Gnemmi V, Leon J, Payet C, Robe-Rybkine T, Paule R, Delguste F, Satchell SC, et al. Heme Drives Susceptibility of Glomerular Endothelium to Complement Overactivation Due to Inefficient Upregulation of Heme Oxygenase-1. *Front Immunol* (2018) **9**: doi: 10.3389/fimmu.2018.03008

2. Sartain SE, Turner NA, Moake JL. TNF Regulates Essential Alternative Complement Pathway Components and Impairs Activation of Protein C in Human Glomerular Endothelial Cells. *J Immunol* (2016) **196**:832–845. doi: 10.4049/jimmunol.1500960

3. Sartain SE, Turner NA, Moake JL. Brain microvascular endothelial cells exhibit lower activation of the alternative complement pathway than glomerular microvascular endothelial cells. *J Biol Chem* (2018) **293**:7195–7208. doi: 10.1074/jbc.RA118.002639

4. Roumenina LT, Jablonski M, Hue C, Blouin J, Dimitrov JD, Dragon-Durey M-A, Cayla M, Fridman WH, Macher M-A, Ribes D, et al. Hyperfunctional C3 convertase leads to complement deposition on endothelial cells and contributes to atypical hemolytic uremic syndrome. *Blood* (2009) **114**:2837–2845. doi: 10.1182/blood-2009-01-197640

5. Lin Y, Weisdorf DJ, Solovey A, Hebbel RP. Origins of circulating endothelial cells and endothelial outgrowth from blood. *J Clin Invest* (2000) **105**:71–77. doi: 10.1172/JCI8071

6. Mcginn S, Poronnik P, Gallery ED, Pollock CA. A method for the isolation of glomerular and tubulointerstitial endothelial cells and a comparison of characteristics with the human umbilical vein endothelial cell model. *Nephrology* (2004) **9**:229–237. doi: 10.1111/j.1440-1797.2004.00254.x

7. Murakami S, Morioka T, Nakagawa Y, Suzuki Y, Arakawa M, Oite T. Expression of Adhesion Molecules by Cultured Human Glomerular Endothelial Cells in Response to Cytokines: Comparison to Human Umbilical Vein and Dermal Microvascular Endothelial Cells. *Microvasc Res* (2001) **62**:383–391. doi: 10.1006/mvre.2001.2356

8. Satchell SC, Tasman CH, Singh A, Ni L, Geelen J, von Ruhland CJ, O’Hare MJ, Saleem MA, van den Heuvel LP, Mathieson PW. Conditionally immortalized human glomerular endothelial cells expressing fenestrations in response to VEGF. *Kidney Int* (2006) **69**:1633–1640. doi: 10.1038/sj.ki.5000277

9. Ades EW, Candal FJ, Swerlick RA, George VG, Summers Susan, Bosse DC, Lawley TJ. HMEC-1: Establishment of an Immortalized Human Microvascular Endothelial Cell Line. *J Invest Dermatol* (1992) **99**:683–690. doi: 10.1111/1523-1747.ep12613748

10. Noone DG, Riedl M, Pluthero FG, Bowman ML, Liszewski MK, Lu L, Quan Y, Balgobin S, Schneppenheim R, Schneppenheim S, et al. Von Willebrand factor regulates complement on endothelial cells. *Kidney Int* (2016) **90**:123–134. doi: 10.1016/j.kint.2016.03.023

11. Louise CB, Obrig TG. Human Renal Microvascular Endothelial Cells as a Potential Target in the Development of the Hemolytic Uremic Syndrome as Related to Fibrinolysis Factor Expression, in Vitro. *Microvasc Res* (1994) **47**:377–387. doi: 10.1006/mvre.1994.1030

12. Du L, Dong F, Guo L, Hou Y, Yi F, Liu J, Xu D. Interleukin-1β increases permeability and upregulates the expression of vascular endothelial-cadherin in human renal glomerular endothelial cells. *Mol Med Rep* (2015) **11**:3708–3714. doi: 10.3892/mmr.2015.3172
