# Supplementary material for: Ex Vivo Test for Measuring Complement Attack on Endothelial Cells: From Research to Bedside
Source: Front Immunol. 2022 Apr 12;13:860689. doi: 10.3389/fimmu.2022.860689 (PMC9041553; doi:10.3389/fimmu.2022.860689)
Supplement: Supplementary file 2 [file Table_2.docx]

**Table S2**: Specific characteristics of the endothelial cells after stimulation

| Characteristics | Endothelial cells |
| --- | --- |
| Behavior after TNF alpha stimulation compared to resting cells | |
| PECAM-1 = CD31 | ↓HMEC, HUVEC and HRGEC (ELISA Cell) (1) |
| VCAM1 | ↑ : HUVEC and HRGEC; no effect HMEC (ELISA Cell) (1) |
| E selectine | ↑HMEC, HRGEC and HUVEC (ELISA Cell) (1) |
| ICAM1 | ↑HMEC1 ≈ HMEC (FACS) (2) |
| tPA (supernatant) | ↓ HUVEC, ↑HUVEC (ELISA) (3) |
| uPA (supernatant) | ↑HUVEC , no effect HRGEC (ELISA) (3) |
| PAI- 1 (supernatant) | ↑HUVEC , ↓ HRGEC (ELISA) (3) |
| Ratio tPA+uPA:PAI1 | Accentuation of HUVEC antifibrinolytique (1:227) vs HRGEC profibrinolytique (75:1) (3) |
| CD46 / CD55 | BMVEC ≈ / x2 > HRGEC (FACS) (4) |
| FH / C3 / FB (supernatant) | BMVEC x 1,5 / x4 / x4 >HRGEC (ELISA) (4) |
| C3a / Ba / C5a generation (supernatant) | BMVEC<<< / >x2 / <<< HRGEC (ELISA) (4) |
| FB cleavage to Ba | HRGEC: 4%; BMVEC : 2% (ELISA) (4) |
| C3aR / C5aR gene expression | BMVEC x23<HRGEC / Undetectable (RTqPCR) (4) |
| C3aR / C5aR protein expression | BMVEC x2 > HRGEC / Undetectable (FACS) (4) |
| CD46 / CD55 / CD59 / TM | Profile HUVEC≈HRGEC: ≈ / ≈ / ≈ / ↓ (FACS) (5) |
| CD46 / CD55 / CD59 / TM gene expression | Profile HUVEC≈HRGEC: ≈ / ≈ / ≈ / ↓ (RTqPCR) (5) ; BMVEC x 4 / x18 / NA/ x10 > HRGEC (RTqPCR)(4) |
| FI / FH gene expression | BMVEC x7 / x23 > HRGEC (RTqPCR)(4); minor effect: HUVEC≈HRGE (RTqPCR) (5) |
| C3 / FB gene expression | ↑ HRGEC > HUVEC (RTqPCR)(5) BMVEC x7 / x4 > HRGEC (RTqPCR) (4) |
| C5 / C4 / FD / FP gene expression | BMVEC x5>/x8 >/x3</x 4< HRGEC (RTqPCR) (4) ; minor effect: HUVEC≈HRGE (RTqPCR) (5) |
| VWF et ADAMTS13 gene expression | Minor effect: HUVEC≈HRGEC (RTqPCR) (5) |
| C3 / FB generation (supernatant) | ↑/ ↑ HUVEC and HRGEC (Fluorescence immunoassay)(5); HUVEC : ↑ x 11 / ↑ x 13 (ELISA) (6) |
| Behavior after INF gamma stimulation compared to resting cells | |
| PECAM-1 = CD31 | ↓HMEC, HUVEC and HRGEC (ELISA Cell) (1) |
| VCAM1 | ↑HUVEC ; ≈ HMEC and HRGEC (ELISA Cell) (1) |
| E selectine | No effect: HMEC, HUVEC and HRGEC (ELISA Cell) (1) |
| FH production (supernatant) | HUVEC:↑ (ELISA) (7) |
| FH expression (mRNA) | HUVEC:↑ ) (Northern Blot) (7) |
| C2, C3, FB, C1inh, FH expression (mRNA) | HUVEC:↑/↓ / ↑/ ↑/ ↑ (Northern Blot) (8) |
| C2, C3, FB, C1inh, FH generation (supernatant) | HUVEC:↑/↓ / ↑/ ↑/ ↑ (ELISA) (8) |
| C3 / FB (supernatant) | HUVEC : ≈/↑ x5,5 (ELISA) (6) |
| C3 / C4 cellular synthesis | HMEC1: ≈/↑ CI-GEnC : ↑ /↑ (WB on lysates) (9) |
| Behavior after IL1 beta stimulation compared to resting cells | |
| VE Cadherin gene and protein expression | ↓HUVEC, ↑ HRGEC (RTqPCR, WB and IF) (10) |
| Permeability * | ↑ HUVEC > HRGEC (10) |
| Trans Endothelial Electrical Resistance ** | ↓ HUVEC > HRGEC (10) |
| CD46 / CD55 / CD59 / TM protein expression | HUVEC & HRGEC : Minor effect (FACS) (5) |
| CD46 / CD55 / CD59 / TM gene expression | HUVEC & HRGEC : Minor effect (RTqPCR) (5) |
| C3 / FB gene expression | ↑C3 and FB HUVEC>HRGEC (RT-qPCR) (5) |
| C5, FI, C4, VWF et ADAMTS13, Properdin, FD, CFH gene expression | No effect : HUVEC≈HRGEC (RT-qPCR) (5) |
| C3 / FB (supernatant) | HUVEC : ↑x4,8 / ≈ (ELISA)(6) |
| Behavior after IL1 alpha stimulation compared to resting cells | |
| C3 / FB / FH generation (supernatant) | HUVEC : ↑/ ↑/↓ (ELISA) (11) |
| C3 / FB expression (mRNA) | HUVEC : ↑/ ↑ (Northern Blot) (11) |
| Behavior after LPS stimulation |  |
| tPA (supernatant) | ↓ HUVEC, ↑HUVEC (ELISA) (3) |
| uPA (supernatant) | ↑HUVEC, no effect HRGEC(ELISA) (3) |
| PAI- 1 (supernatant) | ↑HUVEC, ↓ HRGEC (ELISA) (3) |
| Ratio tPA+uPA:PAI1 | Accentuation of HUVEC antifibrinolytique (1:227) vs HRGEC profibrinolytique (75:1) (3) |
| C3 / C4 cellular synthesis | HMEC-1 and CI-GEnC : ↑/ ≈ (WB on lysates) (9) |
| Behavior after heme exposure, NHS incubation compared to resting cells | |
| C3 deposits (heme overnight exposure) | HRGEC≈GENC>HMEC1≈HUVEC (FACS) (12) |
| C3 deposits (heme overnight exposure and 30min rechallenge) | ↑ CI-GEnC and HRGEC ; ≈ HMEC1 ; ↓ HUVEC (FACS) (12) |
| Binding FH from NHS | ↑ HRGEC≈CI-GEnC<HMEC1≈HUVEC (FACS) (12) |
| MCP Expression | ↓ HRGEC≈CI-GEnC≈HMEC1≈HUVEC (FACS) (12) |
| Thrombomodulin gene expression | ↑ HUVEC > HRGEC≈CI-GEnC≈HMEC1 (RTqPCR) (12) |
| HO-1 gene expression | ↑HUVEC > HRGEC≈CI-GEnC≈HMEC1 (RTqPCR) (12) |
| CD55 / CD59 expression | HUVEC : ↓/ ↓ (FACS) (13) |
| P Selectin expression | HUVEC : ↑(FACS) (13) |
| vWF generation (supernatant) | HUVEC : ↑ (ELISA) (13) |

Abbreviations:

BMVEC: Brain Microvascular Endothelial Cells

BOEC: Blood outgrowth endothelial cells

CI-GEnC: Conditionnally Immortalized Human Glomerular Endothelial Cell

HMEC: Human Microvascular Endothelial Cells

HRGEC: Human Renal Glomerular Endothelial Cell

HUVEC: Human Umbilical Vein Endothelial Cells

**References**

1. Murakami S, Morioka T, Nakagawa Y, Suzuki Y, Arakawa M, Oite T. Expression of Adhesion Molecules by Cultured Human Glomerular Endothelial Cells in Response to Cytokines: Comparison to Human Umbilical Vein and Dermal Microvascular Endothelial Cells. *Microvasc Res* (2001) **62**:383–391. doi: 10.1006/mvre.2001.2356

2. Ades EW, Candal FJ, Swerlick RA, George VG, Summers Susan, Bosse DC, Lawley TJ. HMEC-1: Establishment of an Immortalized Human Microvascular Endothelial Cell Line. *J Invest Dermatol* (1992) **99**:683–690. doi: 10.1111/1523-1747.ep12613748

3. Louise CB, Obrig TG. Human Renal Microvascular Endothelial Cells as a Potential Target in the Development of the Hemolytic Uremic Syndrome as Related to Fibrinolysis Factor Expression, in Vitro. *Microvasc Res* (1994) **47**:377–387. doi: 10.1006/mvre.1994.1030

4. Sartain SE, Turner NA, Moake JL. Brain microvascular endothelial cells exhibit lower activation of the alternative complement pathway than glomerular microvascular endothelial cells. *J Biol Chem* (2018) **293**:7195–7208. doi: 10.1074/jbc.RA118.002639

5. Sartain SE, Turner NA, Moake JL. TNF Regulates Essential Alternative Complement Pathway Components and Impairs Activation of Protein C in Human Glomerular Endothelial Cells. *J Immunol* (2016) **196**:832–845. doi: 10.4049/jimmunol.1500960

6. Kawakami Y, Watanabe Y, Yamaguchi M, Haruko Sakaguchi, Kono I, Ueki A. TNF-α stimulates the biosynthesis of complement C3 and factor B by human umbilical cord vein endothelial cells. *Cancer Lett* (1997) **116**:21–26. doi: 10.1016/S0304-3835(97)04737-X

7. Brooimans RA, van der Ark AA, Buurman WA, van Es LA, Daha MR. Differential regulation of complement factor H and C3 production in human umbilical vein endothelial cells by IFN-gamma and IL-1. *J Immunol Baltim Md 1950* (1990) **144**:3835–3840.

8. Lappin DF, Guc D, Hill A, McShane T, Whaley K. Effect of interferon-γ on complement gene expression in different cell types. *Biochem J* (1992) **281**:437–442. doi: 10.1042/bj2810437

9. Hamer R, Molostvov G, Lowe D, Satchell S, Mathieson P, Ilyas R, Mitchell DA, Lam FT, Kashi H, Tan LC, et al. Human Leukocyte Antigen-Specific Antibodies and Gamma-Interferon Stimulate Human Microvascular and Glomerular Endothelial Cells to Produce Complement Factor C4: *Transplant J* (2012) **93**:867–873. doi: 10.1097/TP.0b013e31824b3762

10. Du L, Dong F, Guo L, Hou Y, Yi F, Liu J, Xu D. Interleukin-1β increases permeability and upregulates the expression of vascular endothelial-cadherin in human renal glomerular endothelial cells. *Mol Med Rep* (2015) **11**:3708–3714. doi: 10.3892/mmr.2015.3172

11. Coulpier M, Andreev S, Lemercier C, Dauchel H, Lees O, Fontaine M, Ripoche J. Activation of the endothelium by IL-1α and glucocorticoids results in major increase of complement C3 and factor B production and generation of C3a. *Clin Exp Immunol* (2008) **101**:142–149. doi: 10.1111/j.1365-2249.1995.tb02290.x

12. May O, Merle NS, Grunenwald A, Gnemmi V, Leon J, Payet C, Robe-Rybkine T, Paule R, Delguste F, Satchell SC, et al. Heme Drives Susceptibility of Glomerular Endothelium to Complement Overactivation Due to Inefficient Upregulation of Heme Oxygenase-1. *Front Immunol* (2018) **9**: doi: 10.3389/fimmu.2018.03008

13. Frimat M, Tabarin F, Dimitrov JD, Poitou C, Halbwachs-Mecarelli L, Fremeaux-Bacchi V, Roumenina LT. Complement activation by heme as a secondary hit for atypical hemolytic uremic syndrome. *Blood* (2013) **122**:282–292. doi: 10.1182/blood-2013-03-489245
