# Supplementary material for: Ex Vivo Test for Measuring Complement Attack on Endothelial Cells: From Research to Bedside
Source: Front Immunol. 2022 Apr 12;13:860689. doi: 10.3389/fimmu.2022.860689 (PMC9041553; doi:10.3389/fimmu.2022.860689)
Supplement: Supplementary file 3 [file Table_3.docx]

**Table S3:** Comparative analysis of different protocols used for the ex vivo complement activation test on endothelial cells.

|  | **Human Microvascular Endothelial Cells**  **(HMEC-1)** | | | **Human Umbilical Vein Endothelial Cells (HUVEC)** | **Blood Outgrowth Endothelial Cells (BOEC)** | | **Conditionnally Immortalized Human Glomerular Endothelial Cell (CI-GEnC)** |
| --- | --- | --- | --- | --- | --- | --- | --- |
| Pre-Activation | | | | | | | |
| Resting cells | (1)(2)(3)(4)(5)(6)(7)(8)(9) | | | (10)(11)(12)(13)(14)(15)(16)(17) | (18) | | (10) (11) (12) (19) |
| ADP | 10 μM, 10min (1) (7) (20)(3)(4)(2)(6)(21) (8) (9) | | |  |  | |  |
| TNF aplha + INF gamma |  | | | (11)(13)(14) |  | | (11) |
| LPS | 100 ng/ml 2h (1) | | |  |  | |  |
| Thrombine | 2U/ml 10min(1) | | |  |  | |  |
| Apoptonecrotic* |  | | | (10)(14) |  | |  |
| Necrotic** |  | | | (10)(22)(23) |  | | (19) |
| Heme |  | | | 20min, 37°C(12)(14)(17)(24) |  | | 20min, 37°C (12) |
| Incubation | |  |  | |  |  | |
| Type of blood product incubated | Serum(1)(7)(20)(4)(2)(6)(21) (9), "Activated Plasma"(3)(5)(25) (8) | | | Serum(11)(12)(13)(15)(23)(17)(24); NHS+IgG 500ùg/ml(16)(22); FB-depleted serum supplemented with mutant FB (10)(14) | Serum (18) | | Serum (11) (12) (19), FB-depleted serum supplemented with mutant FB (10) |
| Dilution | 1:2 (1)(20)(3)(4)(2)(5) (8) (9) | | | 1:4(10)(12); 1:3 (16)(13), 1:10 (22) |  | | 1:4 (10)(12) 1:3 (19) |
| Duration | 4h (1)(20)(3)(5) (8) (9), 3h(2) (7) (6), 4h-ADP et 2h-resting(4) | | | 30 min (10)(12)(16)(22)(17)(24) | 1 h (18) | | 30 min (10) (12) (19) |
| Use of AP complement inhibitors | Anti FB Ab: TA106 150 μg/ml(1) | | | Ox24 = Blocking anti FH Ab(11) | Blocking anti CD59 Ab (18) | | Ox24 = Blocking anti FH Ab(11) |
|  | Blocking anti-properdin Ab(3) | | | GB24 =Blocking anti MCP Ab(11) | Blocking anti CD55 Ab (18) | | GB24 =Blocking anti MCP Ab(11) |
|  | Recombinant human complement Factor H(3) | | | FB depleted serum(12)(24) | Blocking anti CD46 Ab (18) | | FB depleted serum (12) |
|  |  | | | Anti-properdin Ab(24) |  | |  |
| Use of LP and CP complement inhibitors | sCR1(7) | | | EGTA-Mg (10)(12)(16)(22); C2 depleted serum(12) | C1q depleted serum(18) | | EGTA-Mg (10) (1,3) C2 depleted serum (12) |
| Use of AP/LP/CP complement inhibitors | sCR1: TP-10, 150 μg/ml (1)(20) | | | EDTA(12)(24) |  | | EDTA (12) |
| Use of TP complement inhibitor | Blocking human complement C5 minibody (1) (7), Eculizumab (1)(3), Anti-human C7 Ab (1), Avacopan (C5aRA antagonist) (7) | | | SSL7 (secreted by Staph aureus) vs different Single Domain Ab anti C5 (including sdAb_E4)(23); Eculizumab (24) |  | |  |
| Other complement modulation | Addition of : CR2-CFH (TT30 150 or 300 μg/ml; CFH concentrate from human plasma (230 μg/ml) (1) ; wild-type “purified FHR1”(20), Heat inactivated serum(2) | | | Supplementation with different concentrations of purified FH(11)(13) |  | | Supplementation with different concentrations of purified FH (11) |
| Other modulation | Blocking FXII with corethrypsin(3), thrombin with hirudin(3). Anticoagulation with EDTA or LMWH(3) | | | Addition of hemopexin(17) |  | |  |

*apoptotic and necrotic cells : staurosporine-induced

**Necrotic cells: overnight detached HUVECs; annexin V, propidium iodide, and trypan blue positive

**Abbreviations:**

ADPKD: Autosomal Dominant Polycystic Kidney Disease

aHUS : Atypical Hemolytic Uremic Syndrome

APS: AntiPhospholipid Syndrome

C3G: C3 Glomerulopathy

CFZ: Carfilzomib

CKD: Chronic Kidney Disease

DDD: Dense Deposit Disease

GPI: Glycosyl Phosphatidyl Inositol

HELLP: Hemolysis, Elevated Liver Enzymes, and Low Platelet

HHTR: Hyperhemolytic Transfusion Reaction

MM: Multiple Myeloma

NHS: Normal Human Serum

PIPLC: Phosphatidyl Inositol specific Phospho Lipase C

SCD: Sickle Cell Disease

SLE: Systemic Lupus Erythematosus

TMA: Thrombotic MicroAngiopathie

TTP: Thrombotic Thrombocytopenic Purpura

VWF: Von Willebrand Factor

References

1. Noris M, Galbusera M, Gastoldi S, Macor P, Banterla F, Bresin E, Tripodo C, Bettoni S, Donadelli R, Valoti E, et al. Dynamics of complement activation in aHUS and how to monitor eculizumab therapy. *Blood* (2014) **124**:1715–1726. doi: 10.1182/blood-2014-02-558296

2. Timmermans SAMEG, Abdul-Hamid MA, Potjewijd J, Theunissen ROMFIH, Damoiseaux JGMC, Reutelingsperger CP, van Paassen P, on behalf of the Limburg Renal Registry. C5b9 Formation on Endothelial Cells Reflects Complement Defects among Patients with Renal Thrombotic Microangiopathy and Severe Hypertension. *J Am Soc Nephrol* (2018) **29**:2234–2243. doi: 10.1681/ASN.2018020184

3. Palomo M, Blasco M, Molina P, Lozano M, Praga M, Torramade-Moix S, Martinez-Sanchez J, Cid J, Escolar G, Carreras E, et al. Complement Activation and Thrombotic Microangiopathies. *Clin J Am Soc Nephrol* (2019) **14**:1719–1732. doi: 10.2215/CJN.05830519

4. Galbusera M, Noris M, Gastoldi S, Bresin E, Mele C, Breno M, Cuccarolo P, Alberti M, Valoti E, Piras R, et al. An Ex Vivo Test of Complement Activation on Endothelium for Individualized Eculizumab Therapy in Hemolytic Uremic Syndrome. *Am J Kidney Dis* (2019) **74**:56–72. doi: 10.1053/j.ajkd.2018.11.012

5. Blasco M, Martínez‐Roca A, Rodríguez‐Lobato LG, Garcia‐Herrera A, Rosiñol L, Castro P, Fernández S, Quintana LF, Cibeira MT, Bladé J, et al. Complement as the enabler of carfilzomib‐induced thrombotic microangiopathy. *Br J Haematol* (2020) doi: 10.1111/bjh.16796

6. Timmermans SAMEG, Wérion A, Damoiseaux JGMC, Morelle J, Reutelingsperger CP, van Paassen P. Diagnostic and Risk Factors for Complement Defects in Hypertensive Emergency and Thrombotic Microangiopathy. *Hypertension* (2020) **75**:422–430. doi: 10.1161/HYPERTENSIONAHA.119.13714

7. Aiello S, Gastoldi S, Galbusera M, Ruggenenti PL, Portalupi V, Rota S, Rubis N, Liguori L, Conti S, Tironi M, et al. C5a and C5aR1 are key drivers of microvascular platelet aggregation in clinical entities spanning from aHUS to COVID-19. *Blood Adv* (2021)bloodadvances.2021005246. doi: 10.1182/bloodadvances.2021005246

8. Youssef L, Miranda J, Blasco M, Paules C, Crovetto F, Palomo M, Torramade-Moix S, García-Calderó H, Tura-Ceide O, Dantas AP, et al. Complement and coagulation cascades activation is the main pathophysiological pathway in early-onset severe preeclampsia revealed by maternal proteomics. *Sci Rep* (2021) **11**:3048. doi: 10.1038/s41598-021-82733-z

9. Piras R, Iatropoulos P, Bresin E, Todeschini M, Gastoldi S, Valoti E, Alberti M, Mele C, Galbusera M, Cuccarolo P, et al. Molecular Studies and an ex vivo Complement Assay on Endothelium Highlight the Genetic Complexity of Atypical Hemolytic Uremic Syndrome: The Case of a Pedigree With a Null CD46 Variant. *Front Med* (2020) **7**:579418. doi: 10.3389/fmed.2020.579418

10. Roumenina LT, Jablonski M, Hue C, Blouin J, Dimitrov JD, Dragon-Durey M-A, Cayla M, Fridman WH, Macher M-A, Ribes D, et al. Hyperfunctional C3 convertase leads to complement deposition on endothelial cells and contributes to atypical hemolytic uremic syndrome. *Blood* (2009) **114**:2837–2845. doi: 10.1182/blood-2009-01-197640

11. Roumenina LT, Frimat M, Miller EC, Provot F, Dragon-Durey M-A, Bordereau P, Bigot S, Hue C, Satchell SC, Mathieson PW, et al. A prevalent C3 mutation in aHUS patients causes a direct C3 convertase gain of function. *Blood* (2012) **119**:4182–4191. doi: 10.1182/blood-2011-10-383281

12. Frimat M, Tabarin F, Dimitrov JD, Poitou C, Halbwachs-Mecarelli L, Fremeaux-Bacchi V, Roumenina LT. Complement activation by heme as a secondary hit for atypical hemolytic uremic syndrome. *Blood* (2013) **122**:282–292. doi: 10.1182/blood-2013-03-489245

13. Schramm EC, Roumenina LT, Rybkine T, Chauvet S, Vieira-Martins P, Hue C, Maga T, Valoti E, Wilson V, Jokiranta S, et al. Mapping interactions between complement C3 and regulators using mutations in atypical hemolytic uremic syndrome. *Blood* (2015) **125**:2359–2369. doi: 10.1182/blood-2014-10-609073

14. Marinozzi MC, Vergoz L, Rybkine T, Ngo S, Bettoni S, Pashov A, Cayla M, Tabarin F, Jablonski M, Hue C, et al. Complement Factor B Mutations in Atypical Hemolytic Uremic Syndrome—Disease-Relevant or Benign? *J Am Soc Nephrol* (2014) **25**:2053–2065. doi: 10.1681/ASN.2013070796

15. Marinozzi MC, Roumenina LT, Chauvet S, Hertig A, Bertrand D, Olagne J, Frimat M, Ulinski T, Deschênes G, Burtey S, et al. Anti-Factor B and Anti-C3b Autoantibodies in C3 Glomerulopathy and Ig-Associated Membranoproliferative GN. *J Am Soc Nephrol* (2017) **28**:1603–1613. doi: 10.1681/ASN.2016030343

16. Vasilev VV, Noe R, Dragon-Durey M-A, Chauvet S, Lazarov VJ, Deliyska BP, Fremeaux-Bacchi V, Dimitrov JD, Roumenina LT. Functional Characterization of Autoantibodies against Complement Component C3 in Patients with Lupus Nephritis. *J Biol Chem* (2015) **290**:25343–25355. doi: 10.1074/jbc.M115.647008

17. Roumenina LT, Chadebech P, Bodivit G, Vieira‐Martins P, Grunenwald A, Boudhabhay I, Poillerat V, Pakdaman S, Kiger L, Jouard A, et al. Complement activation in sickle cell disease: Dependence on cell density, hemolysis and modulation by hydroxyurea therapy. *Am J Hematol* (2020) **95**:456–464. doi: 10.1002/ajh.25742

18. Noone DG, Riedl M, Pluthero FG, Bowman ML, Liszewski MK, Lu L, Quan Y, Balgobin S, Schneppenheim R, Schneppenheim S, et al. Von Willebrand factor regulates complement on endothelial cells. *Kidney Int* (2016) **90**:123–134. doi: 10.1016/j.kint.2016.03.023

19. Chauvet S, Roumenina LT, Bruneau S, Marinozzi MC, Rybkine T, Schramm EC, Java A, Atkinson JP, Aldigier JC, Bridoux F, et al. A Familial C3GN Secondary to Defective C3 Regulation by Complement Receptor 1 and Complement Factor H. *J Am Soc Nephrol* (2016) **27**:1665–1677. doi: 10.1681/ASN.2015040348

20. Valoti E, Alberti M, Tortajada A, Garcia-Fernandez J, Gastoldi S, Besso L, Bresin E, Remuzzi G, Rodriguez de Cordoba S, Noris M. A Novel Atypical Hemolytic Uremic Syndrome–Associated Hybrid *CFHR1/CFH* Gene Encoding a Fusion Protein That Antagonizes Factor H–Dependent Complement Regulation. *J Am Soc Nephrol* (2015) **26**:209–219. doi: 10.1681/ASN.2013121339

21. Timmermans S, Damoiseaux J, Reutelingsperger C, van Paassen P. More About Complement in the Antiphospholipid Syndrome. *Blood* (2020) doi: 10.1182/blood.2020005171

22. Radanova M, Mihaylova G, Ivanova D, Daugan M, Lazarov V, Roumenina L, Vasilev V. Clinical and functional consequences of anti‐properdin autoantibodies in patients with lupus nephritis. *Clin Exp Immunol* (2020) doi: 10.1111/cei.13443

23. Yatime L, Merle NS, Hansen AG, Friis NA, Østergaard JA, Bjerre M, Roumenina LT, Thiel S, Kristensen P, Andersen GR. A Single-Domain Antibody Targeting Complement Component C5 Acts as a Selective Inhibitor of the Terminal Pathway of the Complement System and Thus Functionally Mimicks the C-Terminal Domain of the Staphylococcus aureus SSL7 Protein. *Front Immunol* (2018) **9**: doi: 10.3389/fimmu.2018.02822

24. Chen JY, Galwankar NS, Emch HN, Menon SS, Cortes C, Thurman JM, Merrill SA, Brodsky RA, Ferreira VP. Properdin Is a Key Player in Lysis of Red Blood Cells and Complement Activation on Endothelial Cells in Hemolytic Anemias Caused by Complement Dysregulation. *Front Immunol* (2020) **11**: doi: 10.3389/fimmu.2020.01460

25. Cid J, Fernández J, Palomo M, Blasco M, Bailó N, Diaz-Ricart M, Lozano M. Hyperhemolytic Transfusion Reaction in Non-Hemoglobinopathy Patients and Terminal Complement Pathway Activation: Case Series and Review of the Literature. *Transfus Med Rev* (2020) doi: 10.1016/j.tmrv.2020.06.002
