# Supplementary material for: Ex Vivo Test for Measuring Complement Attack on Endothelial Cells: From Research to Bedside
Source: Front Immunol. 2022 Apr 12;13:860689. doi: 10.3389/fimmu.2022.860689 (PMC9041553; doi:10.3389/fimmu.2022.860689)
Supplement: Supplementary file 4 [file Table_4.docx]

**Table S4:** Comparative analysis of different controls used for the ex vivo complement activation test on endothelial cells.

|  | **Human Microvascular Endothelial Cells**  **(HMEC-1)** | **Human Umbilical Vein Endothelial Cells (HUVEC)** | **Blood Outgrowth Endothelial Cells (BOEC)** | **Conditionally Immortalized Human Glomerular Endothelial Cell (CI-GEnC)** |
| --- | --- | --- | --- | --- |
| Pooled sample run in parallel | 8 NHS Pooled(1); Healthy plasma and serum pooled (2)(3)(4) ; 10 NHS pooled (5) (6); NHS Pooled(7)(8)(9) |  |  |  |
| Negative control | One healthy control in parallel (10)(11) ; DDD(1), patients with arteriosclerosis(1); 10 healthy pregnant women(2); ADPKD(2); CKD(5), dialysis(5); 35 NHS(5), MM treated with CFZ(3), 10 NHS(12) | AB normal serum(13), FB-depleted serum supplemented with recombinant WT FB(13) 20 NHS(13); 50 NHS(14), NHS(15)(16)(17)(18)(19) (20)(21); IgG from HD(19)(22) | BOEC from healthy donors *vs* BOEC from deficient vWF patients (23) | AB normal serum(13), FB-depleted serum supplemented with recombinant WT FB(13), 10 NHS (13); 50 NHS(14); NHS (15)(24) |
| Positive control | 0(10)(11)(2)(5)(3), aHUS (1)(8), complement asymptomatic-mutation-carriers(12) | 0(13)(19)(22), FH depleted(14)(15)(16)(18), FI depleted(15), NHS + FH blocking Ab(15)(17), NHS+heme(21) | 0 (23) | 0 (13) ; FH depleted serum(14) (15) (24) aSHU serum carrying known FH mutation (24) |
| Background | Incubation with test medium alone (10)(1) | Incubation with test medium alone(16)(19) |  |  |

**Abbreviations:**

ADPKD: Autosomal Dominant Polycystic Kidney Disease

aHUS : Atypical Hemolytic Uremic Syndrome

CKD: Chronic Kidney Disease

MM: Multiple Myeloma

NHS: Normal Human Serum

References

1. Timmermans SAMEG, Abdul-Hamid MA, Potjewijd J, Theunissen ROMFIH, Damoiseaux JGMC, Reutelingsperger CP, van Paassen P, on behalf of the Limburg Renal Registry. C5b9 Formation on Endothelial Cells Reflects Complement Defects among Patients with Renal Thrombotic Microangiopathy and Severe Hypertension. *J Am Soc Nephrol* (2018) **29**:2234–2243. doi: 10.1681/ASN.2018020184

2. Palomo M, Blasco M, Molina P, Lozano M, Praga M, Torramade-Moix S, Martinez-Sanchez J, Cid J, Escolar G, Carreras E, et al. Complement Activation and Thrombotic Microangiopathies. *Clin J Am Soc Nephrol* (2019) **14**:1719–1732. doi: 10.2215/CJN.05830519

3. Blasco M, Martínez‐Roca A, Rodríguez‐Lobato LG, Garcia‐Herrera A, Rosiñol L, Castro P, Fernández S, Quintana LF, Cibeira MT, Bladé J, et al. Complement as the enabler of carfilzomib‐induced thrombotic microangiopathy. *Br J Haematol* (2020) doi: 10.1111/bjh.16796

4. Youssef L, Miranda J, Blasco M, Paules C, Crovetto F, Palomo M, Torramade-Moix S, García-Calderó H, Tura-Ceide O, Dantas AP, et al. Complement and coagulation cascades activation is the main pathophysiological pathway in early-onset severe preeclampsia revealed by maternal proteomics. *Sci Rep* (2021) **11**:3048. doi: 10.1038/s41598-021-82733-z

5. Galbusera M, Noris M, Gastoldi S, Bresin E, Mele C, Breno M, Cuccarolo P, Alberti M, Valoti E, Piras R, et al. An Ex Vivo Test of Complement Activation on Endothelium for Individualized Eculizumab Therapy in Hemolytic Uremic Syndrome. *Am J Kidney Dis* (2019) **74**:56–72. doi: 10.1053/j.ajkd.2018.11.012

6. Aiello S, Gastoldi S, Galbusera M, Ruggenenti PL, Portalupi V, Rota S, Rubis N, Liguori L, Conti S, Tironi M, et al. C5a and C5aR1 are key drivers of microvascular platelet aggregation in clinical entities spanning from aHUS to COVID-19. *Blood Adv* (2021)bloodadvances.2021005246. doi: 10.1182/bloodadvances.2021005246

7. Timmermans SAMEG, Wérion A, Damoiseaux JGMC, Morelle J, Reutelingsperger CP, van Paassen P. Diagnostic and Risk Factors for Complement Defects in Hypertensive Emergency and Thrombotic Microangiopathy. *Hypertension* (2020) **75**:422–430. doi: 10.1161/HYPERTENSIONAHA.119.13714

8. Timmermans S, Damoiseaux J, Reutelingsperger C, van Paassen P. More About Complement in the Antiphospholipid Syndrome. *Blood* (2020) doi: 10.1182/blood.2020005171

9. Piras R, Iatropoulos P, Bresin E, Todeschini M, Gastoldi S, Valoti E, Alberti M, Mele C, Galbusera M, Cuccarolo P, et al. Molecular Studies and an ex vivo Complement Assay on Endothelium Highlight the Genetic Complexity of Atypical Hemolytic Uremic Syndrome: The Case of a Pedigree With a Null CD46 Variant. *Front Med* (2020) **7**:579418. doi: 10.3389/fmed.2020.579418

10. Noris M, Galbusera M, Gastoldi S, Macor P, Banterla F, Bresin E, Tripodo C, Bettoni S, Donadelli R, Valoti E, et al. Dynamics of complement activation in aHUS and how to monitor eculizumab therapy. *Blood* (2014) **124**:1715–1726. doi: 10.1182/blood-2014-02-558296

11. Valoti E, Alberti M, Tortajada A, Garcia-Fernandez J, Gastoldi S, Besso L, Bresin E, Remuzzi G, Rodriguez de Cordoba S, Noris M. A Novel Atypical Hemolytic Uremic Syndrome–Associated Hybrid *CFHR1/CFH* Gene Encoding a Fusion Protein That Antagonizes Factor H–Dependent Complement Regulation. *J Am Soc Nephrol* (2015) **26**:209–219. doi: 10.1681/ASN.2013121339

12. Cid J, Fernández J, Palomo M, Blasco M, Bailó N, Diaz-Ricart M, Lozano M. Hyperhemolytic Transfusion Reaction in Non-Hemoglobinopathy Patients and Terminal Complement Pathway Activation: Case Series and Review of the Literature. *Transfus Med Rev* (2020) doi: 10.1016/j.tmrv.2020.06.002

13. Roumenina LT, Jablonski M, Hue C, Blouin J, Dimitrov JD, Dragon-Durey M-A, Cayla M, Fridman WH, Macher M-A, Ribes D, et al. Hyperfunctional C3 convertase leads to complement deposition on endothelial cells and contributes to atypical hemolytic uremic syndrome. *Blood* (2009) **114**:2837–2845. doi: 10.1182/blood-2009-01-197640

14. Roumenina LT, Frimat M, Miller EC, Provot F, Dragon-Durey M-A, Bordereau P, Bigot S, Hue C, Satchell SC, Mathieson PW, et al. A prevalent C3 mutation in aHUS patients causes a direct C3 convertase gain of function. *Blood* (2012) **119**:4182–4191. doi: 10.1182/blood-2011-10-383281

15. Frimat M, Tabarin F, Dimitrov JD, Poitou C, Halbwachs-Mecarelli L, Fremeaux-Bacchi V, Roumenina LT. Complement activation by heme as a secondary hit for atypical hemolytic uremic syndrome. *Blood* (2013) **122**:282–292. doi: 10.1182/blood-2013-03-489245

16. Schramm EC, Roumenina LT, Rybkine T, Chauvet S, Vieira-Martins P, Hue C, Maga T, Valoti E, Wilson V, Jokiranta S, et al. Mapping interactions between complement C3 and regulators using mutations in atypical hemolytic uremic syndrome. *Blood* (2015) **125**:2359–2369. doi: 10.1182/blood-2014-10-609073

17. Marinozzi MC, Vergoz L, Rybkine T, Ngo S, Bettoni S, Pashov A, Cayla M, Tabarin F, Jablonski M, Hue C, et al. Complement Factor B Mutations in Atypical Hemolytic Uremic Syndrome—Disease-Relevant or Benign? *J Am Soc Nephrol* (2014) **25**:2053–2065. doi: 10.1681/ASN.2013070796

18. Marinozzi MC, Roumenina LT, Chauvet S, Hertig A, Bertrand D, Olagne J, Frimat M, Ulinski T, Deschênes G, Burtey S, et al. Anti-Factor B and Anti-C3b Autoantibodies in C3 Glomerulopathy and Ig-Associated Membranoproliferative GN. *J Am Soc Nephrol* (2017) **28**:1603–1613. doi: 10.1681/ASN.2016030343

19. Vasilev VV, Noe R, Dragon-Durey M-A, Chauvet S, Lazarov VJ, Deliyska BP, Fremeaux-Bacchi V, Dimitrov JD, Roumenina LT. Functional Characterization of Autoantibodies against Complement Component C3 in Patients with Lupus Nephritis. *J Biol Chem* (2015) **290**:25343–25355. doi: 10.1074/jbc.M115.647008

20. Roumenina LT, Chadebech P, Bodivit G, Vieira‐Martins P, Grunenwald A, Boudhabhay I, Poillerat V, Pakdaman S, Kiger L, Jouard A, et al. Complement activation in sickle cell disease: Dependence on cell density, hemolysis and modulation by hydroxyurea therapy. *Am J Hematol* (2020) **95**:456–464. doi: 10.1002/ajh.25742

21. Chen JY, Galwankar NS, Emch HN, Menon SS, Cortes C, Thurman JM, Merrill SA, Brodsky RA, Ferreira VP. Properdin Is a Key Player in Lysis of Red Blood Cells and Complement Activation on Endothelial Cells in Hemolytic Anemias Caused by Complement Dysregulation. *Front Immunol* (2020) **11**: doi: 10.3389/fimmu.2020.01460

22. Radanova M, Mihaylova G, Ivanova D, Daugan M, Lazarov V, Roumenina L, Vasilev V. Clinical and functional consequences of anti‐properdin autoantibodies in patients with lupus nephritis. *Clin Exp Immunol* (2020) doi: 10.1111/cei.13443

23. Noone DG, Riedl M, Pluthero FG, Bowman ML, Liszewski MK, Lu L, Quan Y, Balgobin S, Schneppenheim R, Schneppenheim S, et al. Von Willebrand factor regulates complement on endothelial cells. *Kidney Int* (2016) **90**:123–134. doi: 10.1016/j.kint.2016.03.023

24. Chauvet S, Roumenina LT, Bruneau S, Marinozzi MC, Rybkine T, Schramm EC, Java A, Atkinson JP, Aldigier JC, Bridoux F, et al. A Familial C3GN Secondary to Defective C3 Regulation by Complement Receptor 1 and Complement Factor H. *J Am Soc Nephrol* (2016) **27**:1665–1677. doi: 10.1681/ASN.2015040348
